# Hepatitis B Awareness among Medical Students and Their Vaccination Status at Syrian Private University

**DOI:** 10.1155/2014/131920

**Published:** 2014-11-12

**Authors:** Nazir Ibrahim, Amr Idris

**Affiliations:** ^1^Internal Medicine and Gastroenterology Departments, Syrian Private University, P.O. Box 36822, Damascus, Syria; ^2^Internal Medicine Department, Syrian Private University, Mazzeh Street, P.O. Box 36822, Damascus, Syria

## Abstract

*Background*. Hepatitis B virus (HBV) is a potentially life-threating infection and a well-recognized occupational hazard for health-care workers including medical students.* Methods*. A cross-sectional study was conducted at Syrian Private University (SPU), Faculty of Medicine, to assess the knowledge and awareness about hepatitis B, the status of hepatitis B vaccination, and the reasons for not getting vaccinated among the first- and the fifth-year medical students.* Results*. The present study demonstrates surprising results and raises issues about the high number of medical students that are not vaccinated or not sure about their vaccination status, which puts them at a higher risk of being infected in the future. Another important issue is the medical students' overall knowledge about this life-threating infection. The students have not been totally educated about the gravity of the situation which requires the need of further HBV education. It is highly recommended that SPU provides the HBV vaccine to all nonvaccinated students attending the faculty of medicine at no cost to encourage them to take the HBV vaccine and to reform some of its educational curriculum to effectively limit the hazardous effects of this disease and elaborate on the serious health consequences of HBV.

## 1. Introduction

Hepatitis B infection is a disease of the liver caused by the hepatitis B virus (HBV), which has a partially double-stranded circular DNA and belongs to the family Hepadnaviridae [1, 2]. Hepatitis B is a major public health concern worldwide. Approximately 30% of the world's population has been infected with HBV [3–5], and more than 350 million are chronically infected with HBV and carry high risk for cirrhosis and liver cancer [6]. At least one million people die annually from HBV related chronic liver disease [7].

HBV is transmitted by body fluids, such as blood and serum, and can exhibit vertical transmission from mother to child. Sexual transmission, vertical transmission, and unsafe injections, including intravenous drug use, are the most common routes of infection for HBV [8–12]. Household contact and occupational health-care exposure to blood products and hemodialysis are other risk factors [13–20]. Health-care workers (HCWs) are reported to have the highest occupational risk for HBV infection [21]. There are 35 million HCWs worldwide, and percutaneous injuries have been estimated to result in approximately 66,000 hepatitis B viral infections per year [21]. Data from the United States in the 1990s showed that unvaccinated HCWs had serologic evidence of past or current HBV infection three to five times greater than the general population [22]. In Syria, there is no specific national strategy or guidelines for preventing hepatitis B infection in health-care settings [23].

A survey of the medical students showed that 30% of reported needle stick injuries occurred in the operation room [22].

The clinical manifestations and natural history of HBV infection vary with age. Clinical acute hepatitis B is more frequent in adults than children, and the probability of becoming a chronic carrier of hepatitis B is greater in children than adults: 80–90% of people perinatally infected compared to <5% of infections occurring in adults [24]. People with chronic hepatitis B have a 15% to 25% risk of dying prematurely from HBV related complications [25].

Acute hepatitis B infection is an illness that begins with prodromal symptoms like anorexia, chills, headache, nausea, vomiting, and malaise. Development of jaundice may then occur but is noted in only 30% of all patients with acute infection. Acute hepatitis B is often unrecognized in children younger than five years old.

Chronic infection with the HBV may be either asymptomatic or associated with chronic inflammation of the liver. After 10 years of chronic infection, about 20% of the patients with hepatitis B have progressed to cirrhosis and about 5% have developed HCC [26]. Chronically infected HBV patients have a 15–25% risk of dying prematurely due to HBV-related cirrhosis and HCC [27]. Hepatitis B is estimated to be the cause of 30% of cirrhosis and 53% of HCC worldwide [28]. Also of note, hepatitis B virus has been linked to membranous glomerulonephritis [29]. Given HBV and its ability to affect multiple organ systems including the liver and kidney, chronic infection is of particular concern.

Prevention is the only safe strategy against high prevalence of viral hepatitis. Having enough knowledge and proper attitudes toward this infection is cornerstones of preventing transmission. Medical students have a very important role in preventing the disease by improving the disease knowledge among themselves and the patients they treat. Safe and effective HBV vaccines have been available since 1982. The implementations of mass immunization programs have been recommended by the World Health Organization since 1991. Since its global expanded coverage, the incidence of HBV infection and liver cancer among infants, children, and adolescents has dramatically decreased. Prevention is focused on vaccination of population groups most at risk. Included in these at risk groups are persons working in the health care field. Since the early 1990's, Syria was among the first countries in the Middle East to use the second generation HBV vaccine as an integral part of the national infant immunization programs. However, in Syria, the universal vaccination of adolescents does not have the same implementation success as compared to infant vaccinations. At the Syrian medical universities including SPU, there is no requirement for the students to be hepatitis B vaccinated or to check the hepatitis B immunity status to be admitted to the medical faculty or begin training at the teaching hospitals. However, there is no information provided that the government has established the goal of eliminating hepatitis B or set a strategy that focuses exclusively or primarily on the prevention and control of viral hepatitis [23].

The aims of this study were to assess the knowledge and awareness of HBV infection and estimate the number of first- and fifth-year medical students covered by hepatitis B vaccination.

## 2. Subjects and Methods

A cross-sectional study was conducted by Internal Medicine and Gastroenterology Departments at the Syrian Private University, Damascus, Syria, in February 2014. The study targeted the first- and the fifth-year medical students at the Syrian Private University, Faculty of Medicine. The total number of students enrolled at the time of the study at Syrian Private University in their first medical school year was 120 students and 80 students in their fifth medical school year. The invitation to complete the survey was sent to all the first-year medical students. The overall response was 64 students from the first-year students. Afterward, a proportionate stratified sampling was taken from the fifth-year medical students. Therefore, the overall response from both the first- and fifth-year medial students was 128.

This study was conducted to assess the students' knowledge and awareness about hepatitis B and to assess the number of students covered by hepatitis B vaccine. All students were interviewed using a structured self-completed questionnaire consisting of 20 questions. The questionnaire consisted of five sections: (1) demographic and academic characteristics; (2) HBV knowledge; (3) HBV prevention; (4) personal HBV-related health history; (5) perception of HBV vaccine and vaccination status. The language of instruction at Syrian Private University is English. Therefore, the questionnaire was given in English. All the subjects were interviewed, questions were explained and translated for all the students included in the study; and anonymity was assured. Before the distribution of the questionnaire, the objectives of the study were explained to participants, and they were informed that their participation was voluntary.

### 2.1. Statistical Analysis Used

Data was coded, entered, and analyzed using the Statistical Package for Social Science (SPSS) version 20.0 (SPSS, Chicago, IL, USA).

Ethical approval of this study was received from the Institutional Ethical Review Board of the Faculty of Medicine, Syrian Private University, Damascus, Syria.

## 3. Results

**Figure 1:**
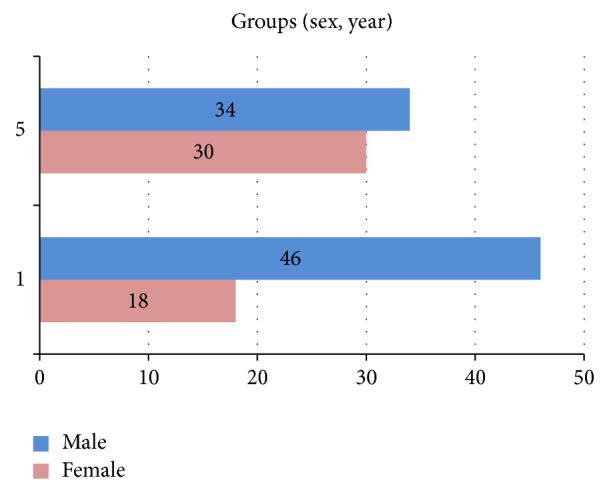
Age groups of the study sample in the first- and fifth-year medicine students.

The demographic characteristics of the study sample are shown in Table 1 and Figure 1. A total of 128 students responded to the questionnaire, 80 (62.5%) males and 48 (37.5%) females. The age of the participants ranged from 17 to 25 years (mean: 21.4). All of the students were from the Faculty of Medicine and were Arabic and English speakers (100%).

The study revealed the weakness of general knowledge about hepatitis B among the junior medical students compared to those in the fifth year. As documented in Table 2, the survey showed that, out of 128 participants who completed the survey, around 92% of subjects are aware of hepatitis B infection, yet unaware of the symptoms, which is significantly associated with the academic level of the students (*P* = 0.000).

The symptoms were well understood by only 37 (57.81%) and 52 (81.25%) of the first- and fifth-year medical students, respectively. In addition, 89.07% of the students did not know that chronic HBV infection is often asymptomatic and only 35.15% of all the students knew that chronic HBV infection confers a high risk of cirrhosis, liver cancer, kidney disease, and its consequences. Understanding of the symptoms and the disease consequences is significantly associated with the medical year of the students (*P* = 0.000).

**Figure 2:**
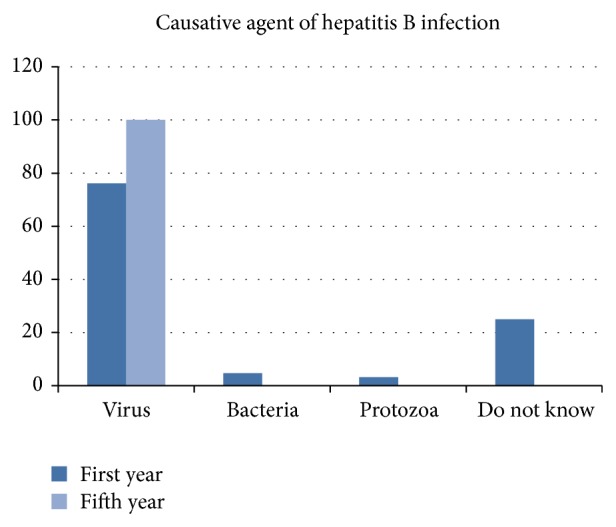
Student's knowledge about the causative agent of hepatitis B infection.

A remarkable difference is found between the students' knowledge of HBV concerning the causative agent and nature (*P* = 0.000), in which the cause of HBV was known to only 43 (67.18%) and 64 (100%) of the first-year and fifth-year students, respectively (Figure 2).

**Figure 3:**
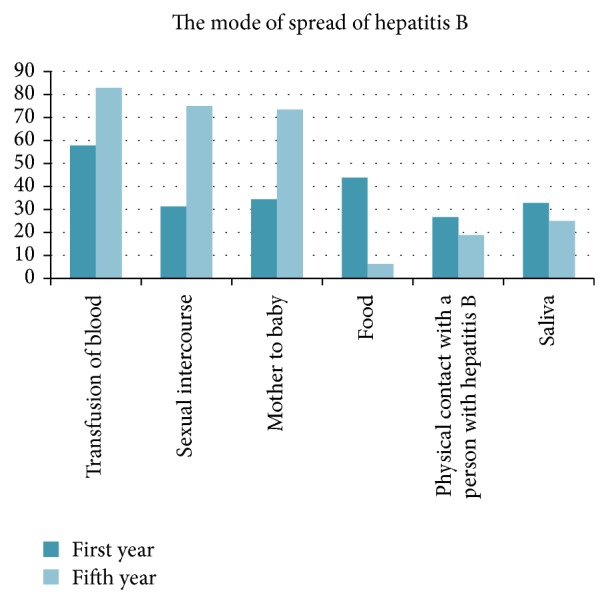
Student's knowledge about the risk factors of hepatitis B infection.

**Figure 4:**
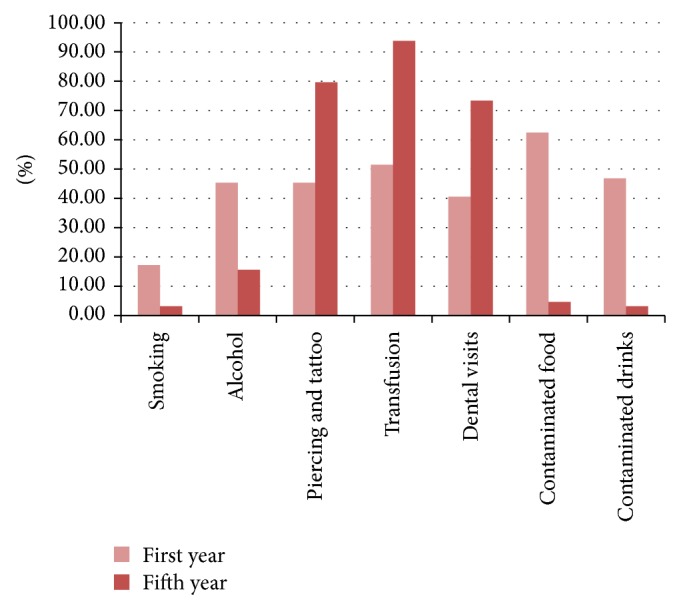
Student's knowledge about routes of transmission of hepatitis B Virus.

Their knowledge about the mode of transmission was also lacking with 52 (40.62%) students unaware that contaminated blood, contaminated needles, unprotected sex with an infected person, and birth to an infected mother are all modes of HBV transmission (*P* = 0.000). Only 25 (39.06%) first-year students and 49 (76.56%) of the fifth-year students are aware of all the modes of HBV transmission (Figure 3). Among 64 first-year students, only one student was aware about all risk factors including piercings, tattoos, transfusion of blood, and dental visits. This number is compared to 33 of the fifth-year students (*P* = 0.000) (Figure 4).

**Figure 5:**
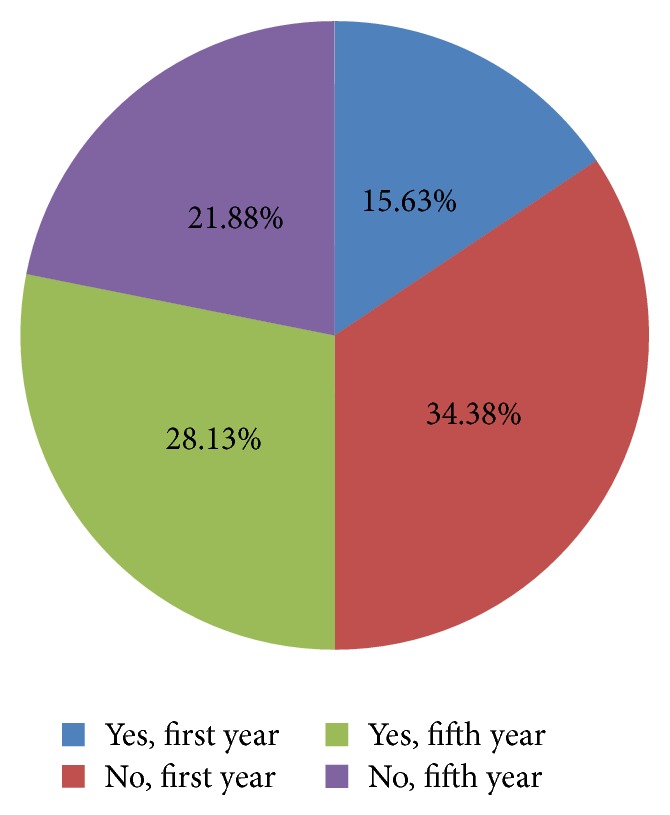
Number of students who received the hepatitis B vaccine.

**Figure 6:**
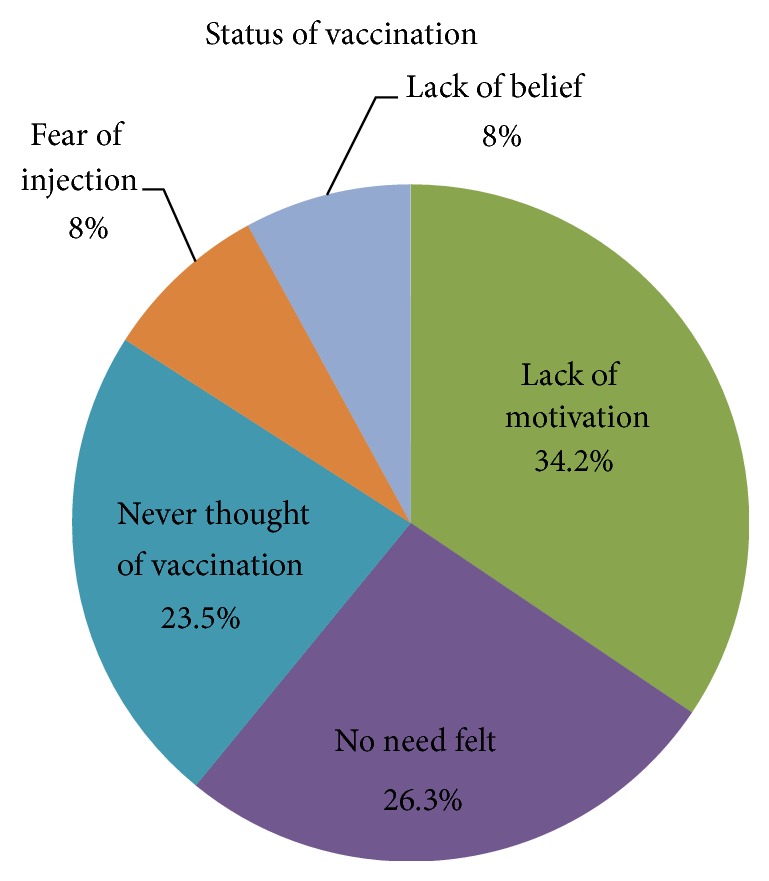
The reason behind not being vaccinated against hepatitis B Virus.

Furthermore, out of the 128 subjects, only 43.75% of the students had taken the hepatitis B vaccine and 26.56% do not know their vaccination status. Of the first-year students, only 20 (31.25%) received the vaccine, compared to 36 (56.25%) of the fifth-year students (*P* = 0.000) (Figure 5). When asked about the reasons of not taking the hepatitis B vaccine, the answers varied between “never thought of vaccination” (23.5%), “lack of motivation” (34.2%), “afraid of needles” (8%), “lack of belief” (8%), and “no need felt” (26.3%), (Figure 6).

Only 16.4% of all the students have gone through the test of HBV infection. Moreover, 12.5% of the individuals did not know that receiving the hepatitis B vaccine and avoiding the reuse of needles are two of the most efficient ways to prevent HBV transmission. 50% of the first-year students are aware that doctors and medical students are more prone of getting hepatitis B via cross-infection while 92.18% of the fifth-year students are aware (*P* = 0.000).

## 4. Discussion

This study showed that the first-year medical students have poor knowledge and lack of awareness about hepatitis B, its routes of transmission, risk factors, and modes of preventions compared to the fifth-year medical students. Similarly, most of the first-year students 63 (98.44%) were not vaccinated against hepatitis B, which makes them vulnerable to the disease. Interestingly, the main reason for not being vaccinated is the lack of motivation (34.2%). However, the survey also shows that most of the students (92%) were aware of hepatitis.

The present study demonstrates surprising results and raises issues about the high number of medical students that are not vaccinated or not sure about their vaccination status. According to a recent study on medical students by Al-Ghamdi, anti-HBs levels were significantly low in many students after their primary immunization. Therefore, testing medical students for anti-HBs levels may be warranted as they represent a high-risk population [30]. Another important issue also rises about the medical students' knowledge about this life-threating infection and the need of further HBV education. Therefore, it is highly recommended that the SPU makes reforms to its educational curriculum to promote awareness among the medical students. One important realization from this study is that education is necessary. As students play an important role in dissemination of knowledge and raising awareness among their communities, more educational efforts should be exerted on the students themselves for the importance of viral hepatitis, and SPU must participate more in national/regional/international meetings about hepatitis in order to contribute to the prevention of viral hepatitis. Furthermore, educational initiatives should also be focused toward avoiding infection and seeking care in case of exposure to infected body fluids.

Another suggestion for a new initiative could be providing free HBV vaccines to all the nonvaccinated students attending medical faculty to encourage universal vaccinations for all students upon their entry. Future studies may be directed at measuring the hepatitis B antibody titers and evaluating the response to the hepatitis B vaccine among the medical students.

## Figures and Tables

**Table 1:** Descriptive characteristics of the subjects included in the analyses.

Characteristics	Number of subjects (%)
Group years	
First year	64 (50%)
Fifth year	64 (50%)
Sex	
Male	80 (62.5%)
Female	48 (37.5%)

**Table 2:** Hepatitis B knowledge questions and correct responses in percentage.

Question	Correct responses (%)	First year (*n* = 64)	Fifth year (*n* = 64)
Have you heard of hepatitis B?	Yes: 92% No: 8%	Yes: 89.06% No: 10.94%	Yes: 96.87% No: 3.13%

Is most Chronic hepatitis b infection (A) symptomatic, (B) asymptomatic?	(A) Symptomatic: 89.07% (B) Asymptomatic: 10.93%	(A) Symptomatic: 84.38% (B) Asymptomatic: 15.62%	(A) Symptomatic: 93.75% (B) Asymptomatic: 6.25%

Are doctors and medical students more prone of getting hepatitis B via cross-infection?	Yes: 71.09% No: 28.91%	Yes: 50% No: 50%	Yes: 92.18% No: 7.82%

Does hepatitis B vaccination protect against the infection?	Yes: 89.84% No: 10.16%	Yes: 90.62% No: 9.38%	Yes: 89.06% No: 10.94%

Have you received hepatitis B vaccine before in Syria or outside Syria?	Yes: 43.75% No: 29.69% Do not know: 26.56%	Yes: 31.25% No: 18.75% Do not know: 50%	Yes: 56.25% No: 40.62% Do not know: 3.13%

What is ideal age of vaccination?	(A) Infancy: 55.46% (B) Youth: 32.03% (C) Adulthood: 12.5%	(A) Infancy: 51.56% (B) Youth: 42.18% (C) Adulthood: 4.68%	(A) Infancy: 59.37% (B) Youth: 21.87% (C) Adulthood: 20.31%

What is the reason behind not being vaccinated?	(A) Lack of motivation: 34.20% (B) No need felt: 26.30% (C) Never thought of vaccination: 23.50% (D) Lack of belief: 8% (E) Fear of injection: 8%	(A) 16.6% (B) 33.33% (C) 25% (D) 16.6% (E) 8.33%	(A) 42.3% (B) 23.07% (C) 23.07% (D) 3.84% (E) 7.69%

Should hepatitis patients be allowed to work?	Yes: 47.65% No: 32.03% Do not Know: 20.31%	Yes: 29.68% No: 46.87% Do not know: 23.43%	Yes: 65.63% No: 17.18% Do not know: 17.18%

What is the causative agent of hepatitis B?	(A) Virus: 83.6% (B) Bacteria: 2.34% (C) Protozoa: 1.56% (D) Do not know: 12.53%	(A) Virus: 67.18% (B) Bacteria: 4.68% (C) Protozoa: 3.12% (D) Do not know: 25%	(A) Virus: 100% (B) Bacteria: 0% (C) Protozoa: 0% (D) Do not know: 0%

What is the mode of spread of hepatitis B?	(A) Transfusion of blood: 70.3% (B) Sexual intercourse: 53.12% (C) Mother to her baby: 53.9% (D) Food: 25% (E) Physical contact with a person with hepatitis B: 22.65% (F) Saliva: 28.9%	(A) 57.81% (B) 31.25% (C) 34.37% (D) 43.75% (E) 26.56% (F) 32.81%	(A) 82.81% (B) 75% (C) 73.43% (D) 6.25% (E) 18.75% (F) 25%

What are the risk factors that may be the cause of hepatitis B?	(A) Smoking: 10.15% (B) Alcohol: 30.46% (C) Piercing and tattoo: 62.5% (D) Blood transfusion: 72.65% (E) Dental visits: 57% (F) Eating from contaminated food: 33.59% (G) Drinking from contaminated drinks: 25%	(A) 17.18% (B) 45.31% (C) 45.31% (D) 51.56% (E) 40.6% (F) 62.5% (G) 46.87%	(A) 3.12% (B) 15.62% (C) 79.68% (D) 93.75% (E) 73.43% (F) 4.68% (G) 3.12%

Do hepatitis B infections cause the following singes and symptoms?	(A) Fever: 19.5% (B) Loss of appetite: 13.28% (C) Nausea: 11.7% (D) Vomiting: 10.15% (E) Jaundice: 22.66% (F) All of the above: 69.53%	(A) 28.12% (B) 17.18% (C) 17.18% (D) 14.06% (E) 32.81% (F) 57.81%	(A) 10.93% (B) 9.37% (C) 6.25% (D) 6.25% (E) 12.5% (F) 81.25%

What does chronic hepatitis B infection lead to?	(A) Liver disease: 48.44% (B) Cirrhosis: 45.31% (C) Kidney disease: 4.69% (D) Liver cancer: 27.34% (E) All of the above: 35.15% (F) None of the above:2.34%	(A) 57.81% (B) 51.56% (C) 7.81% (D) 18.75% (E) 15.62% (F) 4.68%	(A) 39.06% (B) 39.06% (C) 1.56% (D) 35.93% (E) 54.68% (F) 0%

Is hepatitis B infection preventable or not?	Ye: 87.5% No: 12.5%	Yes: 92.18% No: 7.82%	Yes: 82.81% No: 17.19%

Have you ever been tested for hepatitis B?	Yes: 16.4% No: 83.6%	Yes: 21.87% No: 78.13%	Yes: 26.56% No: 73.44%

Have you ever been diagnosed with any liver disease before?	Yes: 15.63% No: 84.37%	Yes: 23.44% No: 76.56%	Yes: 7.82% No: 92.18%
